# Safety, Tolerability, and Immunogenicity of Prescribed Usage of Darbepoetin-Alfa (Hetero Biopharma) in Patients of Chronic Kidney Disease With Renal Anemia: A Post-Marketing Surveillance Study

**DOI:** 10.7759/cureus.14730

**Published:** 2021-04-28

**Authors:** Shubhadeep D Sinha, Vamsi K Bandi, Bala B Reddy, Pankaj Thakur, Sreenivasa Chary, Leela Talluri, Sheejith Kakkunnath

**Affiliations:** 1 Clinical Development and Medical Affairs, Hetero Labs Limited, Hyderabad, IND

**Keywords:** anemia, chronic kidney disease, darbepoetin alfa, post-marketing surveillance

## Abstract

Background

This post-marketing surveillance (PMS), observational, prospective, safety study evaluated the safety, tolerability, and long-term immunogenicity of prescribed usage of Darbepoetin alfa (DA-α, manufactured by Hetero Biopharma, Hyderabad, India) in Indian patients having chronic kidney disease (CKD) with anemia.

Methods

All patients having chronic kidney disease with anemia and prescribed Hetero-Darbepoetin were the target patient population. The present study gathered the data from 503 Hetero-Darbepoetin alfa prescribed patients. This study collected information of patient demography, patient's medical history, concomitant medications, action taken with respect to Hetero-Darbepoetin-alfa, adverse events details (AE term, start date, stop date, severity, action taken, outcome, and causality), periodic hemoglobin (Hb) levels, and abnormal laboratory tests results until treatment is discontinued or the patient is lost to follow-up. Immunogenicity data were collected in 121 patients at the end of treatment and after one year.

Results

Eighty-seven AEs were reported in this study and most of them were mild to moderate in intensity. No deaths or serious adverse events (SAEs) were reported in this study. Anti-drug antibodies were not detected in any subject at the end of the treatment phase and after 12 months long-term follow-up period. The baseline mean hemoglobin value was 8.34 (SD 1.24) g/dL and the last visit mean hemoglobin value was 10.42 ± 1.24 (mean ± SD) g/dL. The mean difference between baseline and last visit in hemoglobin value was 2.10 [2.00, 2.20], statistically significant (p-value <0.0001).

Conclusions

The safety and tolerability of the usage of DA-α are similar to that reported in the published literature of the innovator. No patients showed anti-drug antibodies after treatment. Additionally, the patients also showed significant improvement in hemoglobin levels, compared to baseline.

## Introduction

The primary cause of renal anemia in chronic kidney disease (CKD) is the deficiency of endogenous erythropoietin mainly produced by kidneys [1]. Progression of renal anemia not only increases the risk of cardiovascular (CV) disease but also is an independent risk factor for the deterioration of renal function, causing the vicious cycle known as cardio-renal anemia syndrome [2]. Erythropoietin alfa is the standard of care for the treatment of anemia related to CKD undergoing dialysis and not on dialysis. Darbepoetin alfa (DA-α) has a similar mechanism for erythropoiesis as native and recombinant human erythropoietin (rHuEPO). DA-α is proven to achieve significant reductions in RBC transfusion requirements and clinically relevant improvements in fatigue and other patient-reported outcomes. DA-α has a threefold longer elimination half-life and decreased clearance compared to erythropoietin alfa. This ensures a comparatively reduced number of (DA-α) injections in the treatment of anemia in CKD patients.

Hetero conducted Phase-III interventional clinical studies with DA-α (manufactured by Hetero Biopharma, Hyderabad, India) for obtaining marketing and manufacturing (M & M) approval in India. The study showed efficacy and safety in improving anemia associated with CKD undergoing dialysis and those not on dialysis (CTRI/2012/07/002835) who were administered DA-α for a period of 12-24 weeks [3,4]. The current study was subsequently conducted as an observational, prospective post-marketing surveillance (PMS) study as per the local regulatory requirements to collect real-world safety and long-term immunogenicity data in a prescriber setting.

This article has been published in a Research Square preprint server on November 24, 2020 (https://www.researchsquare.com/article/rs-23193/v2).

## Materials and methods

Study design

This was an observational, multicenter, prospective, non-interventional PMS study performed at nine centers across India between June 21, 2016 (initiation) to September 07, 2018 (termination) to evaluate the safety, tolerability, and long-term immunogenicity of DA-α in daily medical practice conditions. A total of 503 patients were included in this study to evaluate the safety, tolerability, and long-term immunogenicity of DA-α in prescribed settings. Data collection included patient demography, patient's medical history, concomitant medications, action taken with respect to DA-α, AE details, periodic Hb levels, and abnormal laboratory test results. DA-α was dosed either 0.45 mcg/kg once weekly (QW) or 0.75 mcg/kg once every two weeks (Q2W) or 1.5 mcg/kg once monthly, as per regulatory approval and as mentioned in the approved prescribing information. If the increase in Hb was less than 1 g/dL in four weeks, the dose was increased by 25%. If the rise in Hb is greater than 2 g/dL in four weeks, the dose was reduced by 25%. If the Hb exceeds 12 g/dL, a dose reduction was considered. If the Hb continued to increase, the dose was reduced by 25%. If after a dose reduction, Hb continued to increase, the dose was temporarily withheld until the Hb begins to decrease, with reinitiated at 25% lower than the previous dose. Safety, tolerability, and immunogenicity data were recorded in the approved PMS forms.

This study was conducted in accordance with the ethical guidelines outlined in the Declaration of Helsinki, 1964 as revised in 2013, Post Authorization Safety Studies (PMS), as per the guidelines of Schedule Y (amended Drug & Cosmetic Act 2013), and Guidelines for Similar Biologics 2012, India along with subsequent amendments and Indian regulatory laws governing biomedical research in human patients. The study was approved by the Drugs Controller General, India (DCGI), CDSCO and subsequently registered with the clinical trial registry (CTRI/2017/04/008338) retrospectively. Institutional ethics committee approvals were obtained from each participating study center before initiating the study.

Participants

In this study, patients of either gender with CKD suffering from renal anemia prescribed and administered DA-α as per the prescribing information were enrolled. Patients with clinical history/evidence of allergy/hypersensitivity to components of Hetero-Darbepoetin alfa, receiving hormonal agents, therapeutic biologic products, or radiotherapy unless receiving concomitant myelosuppressive chemotherapy were not included as per the prescribing information contraindications.

Study assessments

Adverse events and immunogenicity were assessed during the study. AEs were evaluated based on their expectedness, seriousness, incidence, severity, outcome, duration, action taken, and causality. Immunogenicity was evaluated by assessing serum for the presence of anti-darbepoetin alfa antibodies at baseline, end of treatment (up to 24 weeks), and long-term follow-up after 12 months. Immunogenicity samples were stored in the freezer at −65±10 °C. Anti-darbepoetin alfa antibodies in human serum were detected by acid dissociation bridging ELISA. Samples with an absorbance value (OD) less than the cut-off point were considered negative for anti-drug antibodies. A sample with OD equal to or above the cut-off point was to be considered positive. As a routine, the Hb levels were monitored during the study.

Statistical analysis

This study was planned to include 500 patients prescribed DA-α for anemia associated with CKD during routine clinical practice in India. Baseline summary statistics, including mean, median, and standard deviation for age, weight, and proportion of males/females to be provided by the treatment group. The variables measured on a continuous scale compared using a t-test and the proportion of males/females compared using Fisher’s exact test. AEs were summarized by System Organ Class (SOC) and by preferred terms using the Medical Dictionary for Regulatory Activities Terminology (MedDRA v21). The AE severities were graded by using Common Terminology Criteria for Adverse Events, CTCAE (v4). The causality was assessed by using the WHO-UMC causality assessment system. All statistical analysis was performed using SAS® Version 9.4 (SAS Institute Inc., NC, USA).

## Results

Patient characteristics

A total of 503 patients prescribed DA-α for renal anemia were evaluated for safety, tolerability, and immunogenicity in this PMS study. Of these, 121 patients were also evaluated for immunogenicity at the end of treatment and after one year for long-term immunogenicity. The overall mean age was 50.8 years for males, 49.27 years for females, weight was 60.33 kg for males, 58.19 kg for females, and baseline Hb was 8.34 g/dL in the study participants.

Safety evaluation

A total of 87 patients (17.3%) reported at least one AE during the treatment. Twenty-seven (5.4%) AEs reported in nervous system disorders, 24 (4.8%) AEs reported in general disorders and administration site conditions, 19 (3.8%) AEs reported in gastrointestinal disorders, 9 (1.8%) AEs reported in respiratory, thoracic, and mediastinal disorders, 3 (0.6%) AEs reported in the skin and subcutaneous tissue disorders and 2 (0.4%) AEs reported in musculoskeletal and connective tissue disorders (Table 1). The most frequently reported AEs were headache, reported in 25 (5.0%) patients, pyrexia 13 (2.6%), and pain 7 (1.4%). Three (0.59%) AEs were severe, 22 (4.37%) AEs were moderate, and 62 (12.3%) AEs were mild in intensity. Two (0.4%) AEs were certainly related, 5 (1%) AEs were probably related, 40 (8.0%) AEs were possibly related, 39 (7.8%) AEs were unlikely related, and 1 (0.2%) AE was unclassifiable as assessed by the treating doctor. There were no deaths, life-threatening, and serious adverse events (SAEs) reported in this study.

**Table 1:** List of adverse events that occurred in chronic kidney disease patients treated with DA-α Adverse events are classified by System Organ Class [a] Preferred Term as defined by the Med DRA v21; N = number of patients in specified treatment; n = number of patients at specified category.

Adverse event	Patients treated with DA-αN=503, n (%)
Any treatment-emergent adverse event	87 (17.3)
Gastrointestinal disorders	19 (3.8)
Abdominal distension	1 (0.2)
Abdominal pain	1 (0.2)
Abdominal pain upper	1 (0.2)
Constipation	4 (0.8)
Diarrhea	4 (0.8)
Nausea	5 (1.0)
Vomiting	3 (0.6)
General disorders and administration site conditions	24 (4.8)
Asthenia	1 (0.2)
Chills	1 (0.2)
Malaise	1 (0.2)
Oedema peripheral	2 (0.4)
Pain	7 (1.4)
Pyrexia	13 (2.6)
Investigations	1 (0.2)
Blood pressure abnormal	1 (0.2)
Musculoskeletal and connective tissue disorders	2 (0.4)
Back pain	2 (0.4)
Nervous system disorders	27 (5.4)
Dizziness	1 (0.2)
Headache	25 (5.0)
Pain	1 (0.2)
Psychiatric disorders	3 (0.6)
Autism spectrum disorder	1 (0.2)
Somnolence	2 (0.4)
Respiratory, thoracic, and mediastinal disorders	9 (1.8)
Cough	2 (0.4)
Nasopharyngitis	2 (0.4)
Pain	1 (0.2)
Rhinorrhoea	1 (0.2)
Sneezing	3 (0.6)
Skin and subcutaneous tissue disorders	3 (0.6)
Pruritus	1 (0.2)
Rash	2 (0.4)

Immunogenicity analysis

Out of 503 patients, 121 patients agreed to immunogenicity assessment during the treatment and after long-term follow-up of 12 months. Out of 121 patients, 111 patients had baseline and end of treatment immunogenicity data and 102 patients had baseline, end of treatment, and long-term immunogenicity data after 12 months. No anti-darbepoetin alfa antibodies were detected in this population of patients receiving DA-α at the end of treatment and after 12 months of the follow-up period.

Beneficial effects

**Figure 1:**
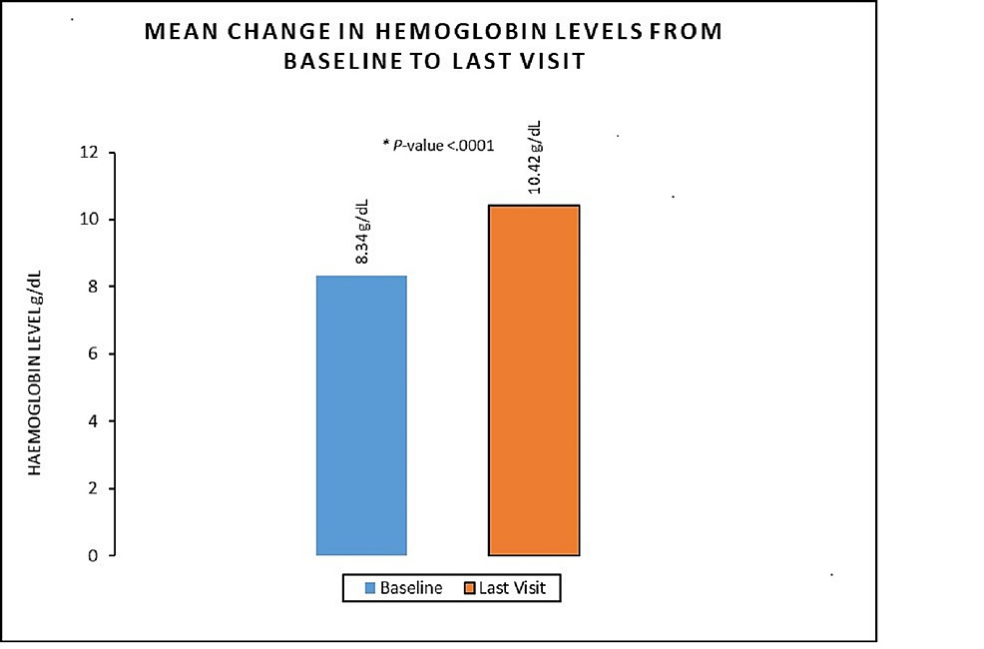
Improvement in the hemoglobin values from the baseline to the last visit by DA-α

The mean Hb levels improved from 8.34 ± 1.24 (mean ± SD) g/dL at baseline to 10.42 ± 1.28 (mean ± SD) g/dL at the end of the treatment (Figure 1). The change in mean Hb levels from baseline to end of treatment was statistically significant [2.10 g/dL (95% CI 2.00, 2.20); p≤0.0001]. 74.95% (377/503) of subjects showed at least 1 g/dL increase in Hb levels at the end of treatment from baseline. Over 61.63% (310/503) of subjects could effectively maintain their Hb levels in the target therapeutic ranges during the treatment period.

## Discussion

Darbepoetin-alfa is the long-acting erythropoietin stimulating agent (ESA) with less frequent dosing intervals compared to epoetins alfa and beta. This study was conducted to evaluate the safety, tolerability, and long-term immunogenicity of DA-α to treat anemia associated with CKD in real-world prescriber settings. Overall, 17.3% of patients reported AEs in this study which is lesser than the phase III study reported AEs (39.7% in dialysis and 25.8% in pre-dialysis) conducted in CKD patients [3,4]. The AEs reported in various SOCs are lesser than the pre-approval studies by DA-α in CKD patients. Respiratory, thoracic, and mediastinal disorders (1.8% vs 14.3%) and general disorders and administration site condition (3.8% vs 12.7%) SOCs reported lesser AEs than the pre-approval studies [3]. Similarly, the most commonly reported AEs like headache (5% vs 9.5%) and pyrexia (2.6% vs 4.8%) reported less frequently in this study compared to previous studies [3-5]. The concern regarding the development of antibodies against darbepoetin leads to loss of its effectiveness negated by negative antibodies at the end of treatment and after one year [5]. Patients who were treated with DA-α could effectively maintain their Hb levels in the target therapeutic ranges during the treatment period and one-year follow-up. The AEs reported in this study were lesser than the popular DREAM-J surveillance study of long-term use of darbepoetin alfa in non-dialysis patients with CKD. DREAM-J study reported 44.4% AEs [6]. This PMS study result suggests that there is no apparent risk for life-threatening or SAEs under clinical conditions where DA-α (manufactured by Hetero Biopharma) is prescribed for the management of anemia associated with CKD.

## Conclusions

Anemia is a frequent complication in patients with CKD and affects the quality of life. Patients with CKD develop uremic anemia as one of the most obvious signs of the disease. DA-α offers an effective treatment of anemia associated with CKD and ameliorates the quality of life. Our study results demonstrate that DA-α is safe and tolerable in treating patients with anemia associated with CKD with significant improvement in hemoglobin levels. These data provide a trend of its safety profile and efficacy in real-world scenario of prescribed settings and is consistent with the phase-III study and published literature.

## References

[1] Babitt JL, Lin HY (2012). Mechanisms of anemia in CKD. J Am Soc Nephrol.

[2] Abrahamson DR, Robert B (2003). Derivation and differentiation of glomerular endothelial cells. Nephrol Dial Transplant.

[3] Sinha SD, Bandi VK, Bheemareddy BR (2019). Efficacy, tolerability and safety of darbepoetin alfa injection for the treatment of anemia associated with chronic kidney disease (CKD) undergoing dialysis: a randomized, phase-III trial. BMC Nephrol.

[4] Mehta KS, Sinha SD, Vamsi B (2019). Darbepoetin alfa versus erythropoietin alfa for treatment of renal anemia in patients with chronic kidney disease at the pre-dialysis stage: a randomized non-inferiority trial. J Assoc Physicians India.

[5] Macdougall IC (2002). Darbepoetin alfa: a new therapeutic agent for renal anemia. Kidney Int Suppl.

[6] Tanaka T, Nangaku M, Imai E (2019). Safety and effectiveness of long-term use of darbepoetin alfa in non-dialysis patients with chronic kidney disease: a post-marketing surveillance study in Japan. Clin Exp Nephrol.

